# Neurodegeneration-induced angioarchitecture remodeling

**DOI:** 10.4103/NRR.NRR-D-25-00408

**Published:** 2025-08-13

**Authors:** Serhii Kostrikov, Torben Moos

**Affiliations:** Centre for Auditory Neuroscience, Hearing Systems Section, Department of Health Technology, Technical University of Denmark, Kgs. Lyngby, Denmark; Neurobiology Research and Drug Delivery, Department of Health Science and Technology, Aalborg University, Aalborg, Denmark

**Studies conducted on neurodegenerative diseases diversely report on changes in the cerebral microvasculature:** Fundamental hallmarks of prevalent neurodegenerative diseases, such as Alzheimer’s disease (AD), dementia with Lewy bodies, Parkinson’s disease, Huntington’s disease, include region-specific neuronal loss and neuroinflammation eventually leading to brain atrophy. Numerous studies have also demonstrated that neurodegeneration and changes in the microvasculature are interconnected. For example, in AD, changes in vessel density have been reported to vary — increasing, remaining unchanged, or decreasing — in brain regions most affected by neurodegeneration. In human cases of Parkinson’s disease, reports have also been contradictory — some suggesting an increase and others suggesting a decrease in vascular density in substantia nigra pars compacta. In mouse models of AD, the vessel density in forebrain areas with neurodegeneration was reported to follow a characteristic pattern with increased density during the early stages of neurodegeneration, which later transformed into stages with lower densities (Thomsen et al., 2025). Considering the very limited effects of amyloid-removal strategies on cognitive decline (van Dyck et al., 2023), interest in the role of vascular pathology in AD pathogenesis is on the rise. Furthermore, relationships between vascular pathology and neurodegeneration are of major interest for conditions of vascular etiology such as cerebral small vessel disease and stroke, as well as the high co-occurrence of the vascular pathology and neurodegenerative diseases.

**Figure 1:**
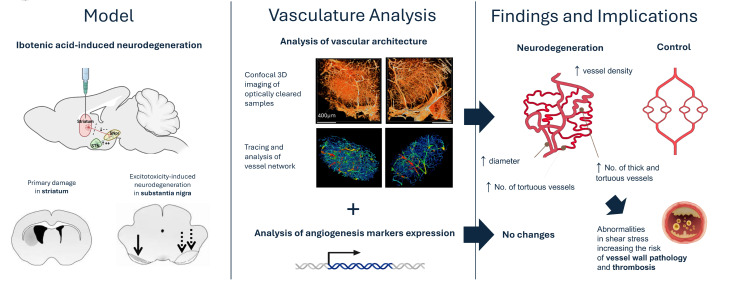
Overview of the recent findings on neurodegeneration-induced angioarchitecture remodeling. (Left) Neurodegeneration is induced by unilateral stereotactic injection of ibotenic acid in the striatum resulting in degeneration of GABAergic neurons (red) projecting to the SNpr. This creates an imbalance between inhibitory inputs from the striatum and excitatory signals from the STN (green), which results in excitotoxicity in SNpr leading to neurodegeneration. After 28 days, there is an evident lesion-induced shrinkage of striatum and the ipsilateral SNpr. These lesions gradually progress, and even more severe shrinkage is seen in the striatum and SNpr 91 days postinduction (single vertical arrow), whereas the opposite side is intact (double vertical arrows). (Middle) Neurodegeneration in substantia nigra pars reticulata is accompanied by changes of the angioarchitecture. 3D rendering of representative confocal images of SNpr and crus cerebri (Crus) from the neurodegeneration (left) and contralateral control (right) sides at long-term (91 days post-surgery). Scale bars in the original images: 400 μm (upper); in preprocessed images: 200 μm (lower). (Right) Schematic depicting possible associations between neurodegeneration-induced angioarchitecture remodeling and lipid accumulation with thrombosis. The observed pattern of angioarchitecture remodeling (increase in diameter, and the number of highly tortuous vessels) is expected to create regions with abnormally low and those with abnormally high shear stress, which in turn increases the risk of pathological depositions, thrombosis, aneurisms formation and endothelial inflammation. This may further exaggerate neurodegeneration, creating a vicious cycle linking the pathogenesis of neurodegeneration and cerebrovascular disease. Reprinted with minor modifications from Thomsen et al. (2025). GABA: Aminobutyric acid; SNpr: substantia nigra par reticulata; STN: subthalamic nucleus.

We recently examined the influence of experimental chronic neurodegeneration on the associated regional microvasculature by combining 3D confocal microscopy of optically cleared samples with vascular tracing and detailed analysis of 3D vessel network architecture (Thomsen et al., 2025). We used an experimental model where neurodegeneration in substantia nigra pars reticulata (SNpr) is caused by excitotoxicity. This was achieved by a microinjection of ibotenic acid into striatum, which led to an imbalance caused by the loss of striatal gamma-aminobutyric acid (GABA) signaling combined with continuous supply of glutamate originating from projections of the subthalamic nucleus (**Figure 1**). The resulting excitotoxity of the striatum triggered substantial pathological changes in the SNpr seven days after injection persisting for at least 3 months. These changes were characterized by clear signs of neurodegeneration and the presence of inflammatory cells, including reactive astrocytes, microglia, and a sustained presence of brain macrophages, which have supposedly migrated into the degenerating tissue (Routhe et al., 2020; Thomsen et al., 2025). Among the most pronounced changes in the SNpr angioarchitecture were an increase in the vessel density (calculated as length of vasculature per tissue volume), an increase in the vessel diameter, and an increase in the number of vessels with high tortuosity. These observations, combined with the negative results of angiogenesis assays, led us to hypothesize that the observed vascular changes result from degeneration of the associated regional brain parenchyma progressing at a faster pace than vascular degeneration (Thomsen et al., 2025). While this is unlikely to impair tissue perfusion — due to the vasculature remaining patent as demonstrated by extensive endothelial binding of wheat germ agglutinin injected into the bloodstream — this geometric pattern of angioarchitecture remodeling increases the risk of thrombosis and development of vessel wall pathologies including aneurysm formation (Heo et al., 2014). Here, we discuss the potential role of such neurodegeneration-induced angioarchitecture remodeling in the pathogenesis of neurodegenerative diseases.

**Driving forces of vessel remodeling and imbalances between glutamate and GABA as a common mechanism for cerebral ischemia and neurodegeneration:** Although the microenvironment of the brain affected by neurodegeneration displays signs of substantial chronic inflammation, and the resulting activation of astrocytes, microglia and invading macrophages clearly provide prerequisites for the enhanced release of pro-angiogenic molecules (Routhe et al., 2020), as was mentioned above, the assays conducted in our study, including endoglin (CD105) detection, did not confirm ongoing angiogenesis in the SNpr. Our imaging data show an increase in the vessel density relative to the associated brain parenchyma without implying absolute vessel volume increase. We also did not detect any classic angioarchitectural signs of angiogenesis such as an increase in the number of blind branches characteristic for sprouting angiogenesis (Gerhardt et al., 2003), which suggests that the observed increase in vessel density is strictly relative and likely results from the vasculature degenerating slower than the associated parenchyma. The purely mechanistic explanation of this observation, i.e. packaging of similar vessel material within a reduced volume, can account for increases in vessel density, tortuosity, and diameter especially when factoring in a decrease in the interstitial pressure, assuming that intravascular pressure remains unchanged. It can also be hypothesized that another contributor to the observed changes is an altered ratio between the vasculature and the cells of the neurovascular unit, which can impair vessel tone and structure.

Keeping the elevated risks of atherosclerosis and atherothrombosis in mind, it can be hypothesized that the vessels eventually may fully occlude thereby making a substantial contribution to the degenerative process occurring in the SNpr. Therefore, our model is unique in that the vascular changes induced by impairing the balance between glutamate and GABA eventually contribute to neurodegeneration remotely from the original lesion side. This has implications for understanding the interface between vascular and neurodegenerative processes in the brain, as regional degeneration like the one occurring in cerebral small vessel disease could affect a particular subset of GABAergic neurons causing excitotoxicity elsewhere in the brain. In a similar way, the identical population of GABAergic neurons could degenerate by non-vascular factors. In both situations, the resulting lack of GABAergic projection could cause excitotoxicity-mediated neural degeneration in a remote brain region, which in turn might even propagate to other downstream regions receiving neuronal projections.

While the neurodegeneration model used in our study relies on excitotoxicity as the sole etiological factor for neurodegeneration, and therefore replicates only certain aspects of the neurodegenerative diseases observed in the clinic, it is important to note that acute ischemic stroke, and possibly also chronic ischemia taking place in cerebral small vessel disease, often provoke dramatic increase in extracellular glutamate due to compromised astrocytic reuptake leading to excessive activation of glutamatergic receptors (Yu et al., 2023). Ranging from modest activation in early AD, the activity of glutamatergic non-synaptic N-methyl-D-aspartate receptors and the associated increase in neuronal sensitivity to glutamate are dramatically elevated in late-stage AD (Yu et al., 2023). This could propagate into swift excitotoxicity causing neuronal Ca^2+^ overload leading to neuronal demise. Shared mechanisms between acute cerebral ischemia and neurodegenerative disorders hence imply a common role of excitotoxicity, leading to increased susceptibility to extracellular glutamate.

It is also important to note that brain capillary endothelial cells express a range of canonical glutamate receptor genes, including the glutamate receptor, ionotropic, N-methyl-D-aspartate (*GRIN*) genes, GRIN1A and GRIN 2A (Peters et al., 2022), which suggest possible direct effects of the excitotoxicity on the endothelium. While our observations do not suggest extensive excitotoxicity-induced degeneration of the endothelium, it is of potential interest to study whether the increased extracellular glutamate also triggers enhanced transport across the blood–brain barrier (BBB) to assist astrocytes in clearing glutamate from the brain extracellular space (O’Kane et al., 1999).

**Potential implications of the observed angioarchitecture changes for the vascular function and BBB permeability:** In our work, we did not focus on examining different aspects of vessel function beyond its patency. However, we can speculate about the potential implications of the inflammatory microenvironment and the downstream effects of the intravascular shear stress caused by angioarchitecture remodeling. Aside from more obvious effects, namely vessel lumen occlusion, ischemia, and disturbed shear stress promote endothelial inflammation (Heo et al., 2014), which can in turn affect the BBB-permeability especially coupled with inflammation in brain parenchyma (Rickman et al., 2023). Among mechanisms leading to an increase in passive BBB permeability in severe cases of cerebral inflammation, migration of macrophages via the paracellular route into the brain can loosen the connections between endothelial cells. However, there are still some controversies remaining about BBB permeability increase in the animal models of neurodegeneration. In good agreement with observations made in experimental animals showing an uncompromised BBB integrity in spite extensive pathology (Bien-Ly et al., 2015), the model examined in our recent study (Thomsen et al., 2025) suggests a conserved expression of molecules important for maintaining the structural integrity of the BBB. However, it remains relevant to test the BBB permeability in this model in a more direct way, for example by tracking the brain parenchyma contents of tracers with different molecular sizes injected into the general circulation. The intertwined signaling pathways of Wnt/β-catenin and nuclear factor-κB were recently addressed in cerebral vessels with pathology. The disturbed blood flow and endothelial inflammation induced by angioarchitecture remodeling can lead to BBB disruption via affecting the signaling of Wnt/β-catenin (Rickman et al., 2023). Impaired astrocytic release of Wnt was shown to increase the BBB to low molecular weight substances (Guérit et al., 2021) and Wnt/β-catenin signaling was inhibited by amyloid-β oligomers (Leasure et al., 2024). The connection between the disturbed flow and upregulation of nuclear factor-κB signaling coupled with reduction in levels of nuclear TAR DNA-binding protein 43, which is important for normal BBB functioning, was well-documented (Omar et al., 2025).

**Remaining questions and future perspectives:** Microvascular remodeling observed in our study raises several questions and opens new avenues for further research:

**·** In terms of therapeutic approaches, our recent findings pose a crucial question: “Can some of the vascular pathology be avoided by preventing neurodegeneration?” A core driver of neurodegeneration in the model we studied is the demise of GABAergic neurons and an increase in glutamatergic signaling. Drugs addressing inhibition of glutamate are already available for treatment of neurodegenerative diseases, including AD and it would be of interest to study their effects on the angioarchitecture.

**·** It is of big interest to experimentally test our hypothesis about the high risk of vessel wall pathology and thrombotic changes in the region undergoing neurodegeneration by applying the ibotenic acid-induced excitotoxicity in animal models with high blood lipid content/hypertension/diabetes. Along the same lines, it remains to be examined if neurodegeneration-induced changes in the angioarchitecture are capable of causing downstream changes in the vessel wall. While we initially hypothesized about the lipid-associated changes, it is important to consider that smaller-caliber vessels are less prone to such pathology and are instead more susceptible to fibrosis and hyalinosis. Our findings have demonstrated that it will be of high interest to develop approaches for modeling of the blood flow in the imaged vessel networks to be able to predict downstream vessel pathology.

**·** It is of relevance to study the role of Wnt/β-catenin signaling for regulating the structural integrity of the remodeling microvasculature under the conditions of chronic neurodegeneration. This could be achieved by combining the ibotenic acid-induced excitotoxicity model with models of Wnt/β-catenin genetic ablation and pharmacological challenges.

**·** In addition to studying vascular remodeling in highly controlled disease models (Thomsen et al., 2025), it is important to examine models that are more representative of the clinical situation (e.g., the 5xFAD AD model) and recapitulate multiple features of the human disease including amyloid deposition and vessel wall impairment.

**·** Glutamate transporters of the brain endothelium have not been thoroughly studied for their transport capacity *in vivo*. Extracellular glutamate increases dramatically in acute stroke (Drejer et al., 1985), presenting relevant experimental settings to examine brain-to-blood transport as a potential compensatory mechanism for the impaired glutamate clearance by ischemic astrocytes.

Ultimately, the pathological human brain affected by chronic neurodegeneration should be examined for the presence of similar vascular changes. The model used in our study demonstrates relatively rapid neurodegeneration, so it is reasonable to ask if similar angioarchitecture changes will take place in case of a substantially slower neurodegeneration. This calls for longitudinal studies, which would allow to establish the sequence of pathogenetic events and shed light on whether the characterization of the vascular profile might serve as a potential diagnostic tool for chronic neurodegenerative/cerebrovascular conditions.

**Conclusions:** The vascular pathology reported in neurodegenerative diseases demands more in-depth research to position the roles of vascular impairment in the picture of disease pathogenesis. Approaches should combine both 3D vessel architecture characterization coupled with blood flow modeling, as well as characterization of expression profiles and function of endothelial surface molecules, particularly neurotransmitter and nutrient transporters amendable for drug delivery purposes.
